# Validation of an electronic frailty index with electronic health records: eFRAGICAP index

**DOI:** 10.1186/s12877-022-03090-8

**Published:** 2022-05-07

**Authors:** Francesc Orfila, Lucía A. Carrasco-Ribelles, Rosa Abellana, Albert Roso-Llorach, Francisco Cegri, Carlen Reyes, Concepción Violán

**Affiliations:** 1 Unitat de Suport a la Recerca Barcelona, Institut Universitari d’Investigació en Atenció Primària Jordi Gol (IDIAP Jordi Gol), Gran Via Corts Catalanes 587, Àtic, 08007 Barcelona, Spain; 2 Gerència Territorial de Barcelona, Institut Català de la Salut, Barcelona, Spain; 3 Institut Universitari d’Investigació en Atenció Primària Jordi Gol (IDIAP Jordi Gol), Barcelona, Spain; 4 Department of Clinical Foundations, Faculty of Medicine, Barcelona University, Barcelona, Spain; 5 Programa de Doctorat en Metodologia de la Recerca Biomèdica i Salut Pública, Universitat Autònoma de Barcelona, Bellaterra, (Cerdanyola del Vallès), Spain; 6 Centre d’Atenció Primària Sant Martí de Provençals, Gerència Territorial de Barcelona, Institut Català de la Salut, Barcelona, Spain; 7 GREMPAL Research Group, CIBERFes and Idiap Jordi Gol, Instituto de Salud Carlos III and Universitat Autonoma de Barcelona, Barcelona, Spain; 8 Unitat de Suport a la Recerca Metropolitana Nord, Institut Universitari d’Investigació en Atenció Primària Jordi Gol (IDIAP Jordi Gol), Mataró, Spain; 9 Universitat Autònoma de Barcelona, Cerdanyola del Vallès, Spain

**Keywords:** Cohort Studies, Electronic Health Records, Frail Elderly, Geriatric Assessment, Primary Health Care, Survival Analysis, Validation Study

## Abstract

**Objective:**

To create an electronic frailty index (eFRAGICAP) using electronic health records (EHR) in Catalunya (Spain) and assess its predictive validity with a two-year follow-up of the outcomes: homecare need, institutionalization and mortality in the elderly. Additionally, to assess its concurrent validity compared to other standardized measures: the Clinical Frailty Scale (CFS) and the Risk Instrument for Screening in the Community (RISC).

**Methods:**

The eFRAGICAP was based on the electronic frailty index (eFI) developed in United Kingdom, and includes 36 deficits identified through clinical diagnoses, prescriptions, physical examinations, and questionnaires registered in the EHR of primary health care centres (PHC). All subjects > 65 assigned to a PHC in Barcelona on 1st January, 2016 were included. Subjects were classified according to their eFRAGICAP index as: fit, mild, moderate or severe frailty. Predictive validity was assessed comparing results with the following outcomes: institutionalization, homecare need, and mortality at 24 months. Concurrent validation of the eFRAGICAP was performed with a sample of subjects (*n* = 333) drawn from the global cohort and the CFS and RISC. Discrimination and calibration measures for the outcomes of institutionalization, homecare need, and mortality and frailty scales were calculated.

**Results:**

253,684 subjects had their eFRAGICAP index calculated. Mean age was 76.3 years (59.5% women). Of these, 41.1% were classified as fit, and 32.2% as presenting mild, 18.7% moderate, and 7.9% severe frailty. The mean age of the subjects included in the validation subsample (*n* = 333) was 79.9 years (57.7% women). Of these, 12.6% were classified as fit, and 31.5% presented mild, 39.6% moderate, and 16.2% severe frailty. Regarding the outcome analyses, the eFRAGICAP was good in the detection of subjects who were institutionalized, required homecare assistance, or died at 24 months (c-statistic of 0.841, 0.853, and 0.803, respectively). eFRAGICAP was also good in the detection of frail subjects compared to the CFS (AUC 0.821) and the RISC (AUC 0.848).

**Conclusion:**

The eFRAGICAP has a good discriminative capacity to identify frail subjects compared to other frailty scales and predictive outcomes.

**Supplementary Information:**

The online version contains supplementary material available at 10.1186/s12877-022-03090-8.

## Background

In most aging societies managing frailty has become a priority for health systems [1, 2]. Defined as a decrease in physiological reserve, frailty puts older individuals at risk of adverse outcomes such as falls, functional decline, institutionalization, and death [3]. Whilst clinical assessment based on history and examination is crucial, and often claimed to be the most accurate approach, it is also subject to variability and the individual sensitivity of the health professional involved.

As a consequence, a number of standardized tools have been developed. The most popular and widely validated is the frailty phenotype described by Fried [4] based on five physical frailty criteria (weight loss, exhaustion, low physical activity, slowness, and weakness). Other scales include multidimensional aspects and risk factors, execution tests such as the Short Physical Performance Battery [5], and laboratory parameters (ghrelin and inflammatory markers among others) [6, 7].

The accumulation of deficit perspective developed by Rockwood and Mitnitski [8] is also widely employed. Its frailty index (FI) is usually considered as the number of deficits an individual has and is calculated as a ratio of deficits present to the total number of deficits considered. The basic principle is that the higher the number of health deficits (diseases, disabilities, symptoms, signs, laboratory, radiographic/electrocardiographic abnormalities) the greater the likelihood of frailty. Deficits chosen when creating a FI must be associated with health status and cover a range of systems. They generally increase with age and should not saturate too early [9]. The FI has been employed in various ways although it needs to be based mainly on aging databases where health deficits are routinely collected.

In most clinical settings frailty measurement is hindered by lack of time, space, and appropriate tools. All too often it is neglected in favour of other health priorities. A valid, standardized, automated FI would overcome such difficulties. It could be used to promote early detection, stratifying and discriminating when there is a need for more extensive assessments and the implementation of effective interventions. Such measures could prevent/delay the onset of frailty, functional decline, and adverse outcomes thus improving quality of life for the elderly. Moreover, stratification would rationalize care cost through the correct allocation of health and social resources.

An automated approach, an electronic FI (eFI), employing electronic health records from primary healthcare databases [10] has been developed in the United Kingdom. Made freely available to primary care as part of general practice [11], the eFI has proven its feasibility and is being replicated in other countries where electronic clinical records exist [12–15].

The objective of this study is to adapt an electronic FI for individuals aged 65 ≥ years using primary care electronic health data from the Catalan Health Care system. In addition, we aim to validate the resulting eFRAGICAP (Electronic Frailty Index, primary healthcare centres, Catalunya) cross-sectionally against other frailty scales, and prospectively predict adverse outcomes such as entry into homecare programs, institutionalization, or death in a two-year follow-up.

## METHODS

### Design, setting, and study population

A longitudinal study was conducted in Barcelona (Spain), a city in the Mediterranean region with 1,604,555 inhabitants. The Spanish National Health Service provides universal coverage, financed mainly by tax revenue. The Catalan Health Institute (ICS) manages 287 primary healthcare centres (PHC) that serve 5,564,292 patients (77.6% PHC) who represent 74% of the population.

A total of 253,684 individuals were included at baseline and 235,670 were alive at the end of the two-year follow-up. Inclusion criteria consisted of individuals aged ≥65 years assigned to a PHC on 1st January 2016. No new entries were allowed. Attrition was caused by mortality or dropouts due to transfer out of the catchment area. During follow-up 5275 individuals moved out of the catchment area (2.08% of the study cohort, with a median follow-up of 347 days).

The cross-sectional validation sample was composed of a random subsample of 333 patients ≥65 years drawn from the cohort and assigned to two PHC. Participants signed an informed consent and none of them were in a homecare programme or institutionalized. They were interviewed in January 2016 to test concurrent validity against two frailty scales, The Clinical Frailty Scale (CFS) [16] and The Risk Instrument for Screening in the Community (RISC) [17], with a cross sectional study in the first month at baseline.

### Data source/s

Data were extracted from the Information System for the Development of Research in Primary Care (SIDIAP) (www.sidiap.org). The SIDIAP database provides anonymized, clinical information coded by family doctors and nurses in the electronic health records (EHR) for the 287 PHC in Catalunya, Spain. The representativeness of the SIDIAP database for the Catalan population in general has already been reported [18, 19]. It contains information on socio-demographics, PHC consultations, referrals, diagnostic codes using the 10th edition of the International Classification of Diseases (ICD-10), clinical/laboratory measures, and other exhaustive clinical information such as geriatric assessments (functional, mobility, cognitive, and social assessments). For this study, we selected all active diagnoses registered in the EHR on January 1st, 2016, and the last values recorded in the other clinical tests or scales. The concurrent subsample interviews were obtained by healthcare professionals blinded to the eFRAGICAP score.

### eFRAGICAP: the frailty index

The eFRAGICAP, based on the accumulation of deficits, was adapted from the eFI [10], and a first version developed using the SIDIAP database [20]. The research team identified all the potential codes from the EHR database that matched the 36 predefined deficits. They included active prescriptions, 50 clinical variables (e.g., scales and laboratory/radiographic values), and 1656 ICD-10 diagnostic codes (Table S1). Deficits were tested following previously described criteria [9], and, as their prevalence logically increased with age, did not reach saturation too quickly.

Frailty status for the eFRAGICAP was obtained by categorising the index into four strata: fit, mild, moderate, or severe according to predefined eFI quartiles. Patients with an eFRAGICAP score of 0–0.12 were defined as fit; > 0.12–0.24 as presenting mild frailty; > 0.24–0.36, as moderate frailty, and > 0.36 as severe frailty. It was then calculated for all the elderly inhabitants of Barcelona assigned to a PHC based on registered clinical information on 1st January 2016.

### Predictive validation outcomes

Three outcomes were studied: all-cause mortality, institutionalization, and inclusion in a homecare program. Mortality was measured using the date of registered death in the PHC records and the Catalan death register. For institutionalization, all types of installations were included (nursing homes and long-term care establishments, either private or public). These are registered in the EHR (ICD-10 code Z59.3) as the care relationship is usually transferred from the PHC to the corresponding institution. For institutionalization prediction, only those non-institutionalized at commencement of follow-up were considered (96.9% of the sample, *N* = 245,923). Inclusion in a PHC homecare programme, either at the request of the patient or healthcare professional due to problems of mobility, is also registered in the EHR (ICD-10 code Z74). For homecare prediction, only those not receiving it or non- institutionalized at commencement of follow-up were included (91.4% of the sample, *N* = 231,763).

### Concurrent validation scales

Concurrent validity was assessed in a random subsample of 333 individuals with two instruments: the CFS and the RISC. The former is a validated measure of frailty based on clinical descriptors and pictographs [16]. Scores range from one (fit) to seven (severe frailty), an individual is considered frail if the CFS is > 4 [21].

The latter records the presence and magnitude of concern across three domains: mental and medical statuses, and Activities of Daily Living (ADLs). Based upon the severity of concerns, and the caregiver network ability to manage them, an overall, global, subjective assessment of risk is then assigned to three adverse outcomes: institutionalization, hospitalization, and death at 1 year from the date of assessment. A simple Likert scale scores five levels of risk from one (minimal and rare) to five (extreme and certain) [17]. An individual is considered at risk if given ≥3 in any adverse outcome.

### Statistical analyses

Continuous variables were expressed as mean and standard deviation (SD) or median and interquartile range (IQ) when appropriate. The effect of the baseline index on mortality prediction was estimated using a Cox regression model. For homecare need and institutionalization outcomes, death before these events was considered a competing risk event, and the cumulative incidence functions (CIF) were calculated. To analyze the effect of the baseline index for the CIF the Fine–Gray regression model [22] for sub-distribution risk (sHR) was employed. Age and gender were included in the multivariate models. The proportionality assumption of the models was verified with the residuals of the models. The models’ discriminative capacity was evaluated by Harrell’s C index [23]. The internal validity of the final predictive models was tested with 100 bootstrap re-samples. Model calibration was verified by plotting the observed and predicted probabilities in groups defined by the deciles of the predicted event probabilities. To measure overall performance of the models, McFadden pseudo-R square was used. Discrimination and calibration were calculated at 1 and 2 years with the pec package [24].

Concurrent validity was studied considering CFS and RISC as gold standard with the previously mentioned cut-off points. Multivariate logistic models were fitted. Discrimination was assessed with receiver operating characteristic curves to estimate areas under the curve (AUC) of the eFRAGICAP for the CFS and RISC. AUC and c-statistics can be considered equivalent measures. Pseudo-R square was also calculated. All analyses were performed with R version 4.1.1.

## RESULTS

The population aged ≥65 years registered at PHC in Barcelona on 1st January 2016 was composed of 253,684 individuals. Global mean age was 76.3 (SD: 7.9) and 59.5% were female, Table 1 depicts the baseline characteristics of the study cohort and the concurrent subsample.

**Table 1:** Characteristics of the study cohort and the concurrent subsample

	Study Cohort (*N* = 253,684)	Concurrent subsample (*N* = 333)
Age (years)	76.3 (7.9)	79.9 (5.9)
Gender: female	59.5%	57.7%
eFRAGICAP score		
Mean (SD)	0.17 (0.12)	0.26 (0.12)
Median (IQR)	0.14 (0.08–0.25)	0.25 (0.18–0.33)
99th percentile	0.52	0.60
Males	0.16 (0.11)	0.25 (0.10)
Females	0.18 (0.12)	0.27 (0.13)
Frailty categories		
Fit	41.1%	12.6%
Mild	32.2%	31.5%
Moderate	18.7%	39.6%
Severe	7.9%	16.2%
Number of comorbidities (*)	2.03 (1.28)	2.70 (1.20)
Number of medications	5.51 (4.0)	8.26 (3.90)
Social deprivation		
1 (least deprivation)	36.9%	0%
2	32.0%	87.1%
3	5.3%	0.6%
4	10.6%	9.3%
5 (most deprived)	15.3%	3.0%
CFS > 4		19.2%
RISC level medium high		23.7%

*Note*: Numeric variables are described as mean (standard deviation) unless otherwise stated. Categorical variables are presented as percentages. (*) out of a list of conditions including cardiovascular disease, cancer, asthma, COPD, diabetes, psychiatric disorders, Parkinson disease, arthritis, and osteoporosis

The global average eFRAGICAP score was 0.17 (SD: 0.12), with a range from 0 to 0.75. Frailty distribution according to eFI reference categorization was 41.1% fit, 32.2% presenting mild frailty, 18.7% moderate frailty, and 7.9% severe frailty. Table S2 presents the prevalence of each of the 36 deficits compared to that of Clegg et al. (original article). The concurrent analysis subsample (*n* = 333) was older, had a mean age 79.9 years (SD: 5.9), and was frailer with an average index of 0.26 (SD: 0.12).

Table 2 depicts the bivariate associations between baseline frailty categories and the three outcomes in the 2-year follow-up. Frailty was significantly associated with death, ranging from 2.1% among the fit to 29.5% for the severe frailty individuals. The same pattern was found in the institutionalization and homecare outcomes, with a range from 0.3 and 0.7%, respectively, in the fit category to 9.5 and 19.2%, respectively, in the severe frailty one. Table 2 also shows the distribution of death before the event (competing risk) for institutionalization and homecare outcomes.

**Table 2:** Description of the study cohort in terms of gender, age, and frailty according to their outcome

	Mortality			Institutionalization				Homecare			
	No event (*N* = 235,670)	Event (*N* = 18,014)	*p*-value	Censored (*N* = 227,116)	Death (*N* = 13,732)	Event (*N* = 5075)	*p*-value	Censored (*N* = 214,871)	Death (*N* = 8771)	Event (*N* = 8121)	*p*-value
Gender			< 0.001				< 0.001				< 0.001
Female	141,293 (93.6%)	9628 (6.4%)		134,974 (93.0%)	6764 (4.7%)	3392 (2.3%)		126,090 (93.4%)	3849 (2.8%)	5084 (3.8%)	
Male	94,377 (91.8%)	8386 (8.2%)		92,142 (91.4%)	6968 (6.9%)	1683 (1.7%)		88,781 (91.8%)	4922 (5.1%)	3037 (3.1%)	
Age	75.7 (7.6)	83.8 (8.2)	< 0.001	75.4 (7.5)	82.7 (8.3)	84.7 (6.8)	< 0.001	74.9 (7.1)	80.6 (8.0)	84.1 (6.6)	< 0.001
Frailty categories			< 0.001				< 0.001				< 0.001
Fit	102,148 (97.9%)	2192 (2.1%)		101,668 (97.7%)	2082 (2.0%)	358 (0.3%)		101,380 (97.5%)	1948 (1.9%)	687 (0.7%)	
Mild	77,961 (95.3%)	3846 (4.7%)		76,144 (94.6%)	3299 (4.1%)	1015 (1.3%)		74,589 (93.7%)	2891 (3.6%)	2161 (2.7%)	
Moderate	41,389 (87.3%)	6047 (12.7%)		37,534 (85.7%)	4238 (9.7%)	2026 (4.6%)		32,734 (83.9%)	2750 (7.1%)	3527 (9.0%)	
Severe	14,172 (70.5%)	5929 (29.5%)		11,770 (67.0%)	4113 (23.4%)	1676 (9.5%)		6168 (67.8%)	1182 (13.0%)	1746 (19.2%)	
eFRAGICAP score	0.164 (0.12)	0.304 (0.14)	< 0.001	0.159 (0.11)	0.288 (0.15)	0.313 (0.13)	< 0.001	0.147 (0.10)	0.229 (0.13)	0.284 (0.12)	< 0.001

Table 3 shows bivariate associations in the concurrent analysis subsample. Individuals rated mildly frail or more in the CFS had a mean eFRAGICAP score of 0.36, while that of those in the first four CFS categories was 0.24. Those who presented medium/high risk in the RISC tool had a mean eFRAGICAP score of 0.38 vs 0.23 of the low-risk subjects.

**Table 3:** Description of the concurrent subsample in terms of gender, age, and frailty according to their classification in the CFS and RISC scales

	Clinical Frailty Scale (CFS)			Risk Instrument for Screening in the Community (RISC)		
	Non-Frail (CFS ≤4) (*N* = 269)	Frail (CFS > 4) (*N* = 64)	*p*-value	Low level (*N* = 254)	Medium-high level (*N* = 79)	*p*-value
Gender			0.01			0.88
Female	146 (76.0%)	46 (24.0%)		147 (76.6%)	45 (23.4%)	
Male	123 (87.2%)	18 (12.8%)		107 (75.9%)	34 (24.1%)	
Age	79.0 (5.5)	84.1 (5.9)	< 0.001	79.1 (5.5)	82.7 (6.3)	< 0.001
Frailty categories			< 0.001			< 0.001
Fit	41 (97.6%)	1 (2.4%)		40 (95.2%)	2 (4.8%)	
Mild	99 (94.3%)	6 (5.7%)		98 (93.3%)	7 (6.7%)	
Moderate	98 (74.2%)	34 (25.8%)		103 (78.0%)	29 (22.0%)	
Severe	31 (57.4%)	23 (42.6%)		13 (24.1%)	41 (75.9%)	
eFRAGICAP score	0.239 (0.11)	0.359 (0.11)	< 0.001	0.227 (0.10)	0.375 (0.12)	< 0.001

**Fig. 1:**
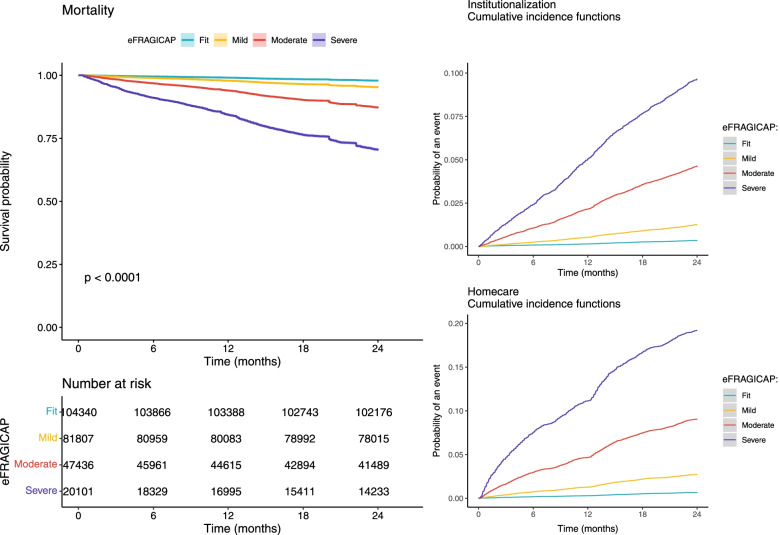
Survival function for mortality and cumulative incidence functions for institutionalization and homecare need. Note: All functions are categorized according to the eFI reference categories

The multivariate analyses are depicted in Table 4. Survival models were used in the predictive validation outcomes, showing adjusted hazard ratios (HR), and accounting for competing risk of death in the case of institutionalization or homecare initiation. Risk of dying rose with every increase in frailty (adjusted HR of 1.69, 3.42, and 7.09 for mild, moderate, and severe frailty, respectively). Institutionalization and homecare need models showed even greater increasing trends. Moderate/severe frailty reported six and more than ten-fold increased risk of institutionalization or homecare initiation, respectively, compared to the fit group. Survival function for mortality and CIF for homecare need and institutionalization are shown in Fig. 1.

**Table 4:** Multivariate models. Baseline frailty, age and gender and death, institutionalization and homecare need at 2 years of follow-up, and concurrent CFS and RISC scales

	Mortality (*N* = 253,684)	Institutionalization (*N* = 245,923)	Homecare (*N* = 231,763)	CFS > 4 (*N* = 333)	RISC Medium-High (*N* = 333)
	HR (95%CI)^a^	sHR (95%CI)^b^	sHR (95%CI)^b^	OR (95%CI)^c^	OR (95%CI)^c^
**Gender: male**	1.85 (1.80–1.91)	0.99 (0.93–1.05)	1.09 (1.04–1.14)	0.56 (0.29–1.09)	2.33 (1.19–4.57)
**Age (years)**	1.09 (1.08–1.09)	1.09 (1.09–1.10)	1.13 (1.12–1.13)	1.13 (1.07–1.20)	1.07 (1.01–1.13)
**Frailty**					
Mild	1.69 (1.61–1.78)	2.66 (2.35–3.00)	2.85 (2.61–3.10)	2.41 (0.27–21.12)	1.18 (0.23–6.04)
Moderate	3.42 (3.25–3.61)	6.63 (5.88–7.47)	6.50 (5.96–7.08)	9.29 (1.20–72.03)	4.09 (0.90–18.52)
Severe	7.09 (6.72–7.49)	10.41 (9.16–11.83)	11.60 (10.54–12.76)	16.67 (2.06–134.98)	58.72 (11.91–289.54)

*Note*: ^a^Cox regression; ^b^Fine-Gray competing risk survival model; ^c^Logistic regression model

The concurrent multivariate models also demonstrated a growing tendency of belonging to the CFS and RISC risk categories as the frailty index increased: adjusted OR for CFS > 4 of 2.41, 9.29, and 16.67, and adjusted OR for RISC medium-high of 1.18, 4.09, and 58.72 for mild, moderate, and severe frailty, respectively.

Table 5 shows the discriminative capacity of the different models and the internal validity of the final predictive models (mortality, institutionalization, and homecare). C-statistics at one- and two-year follow-up are 0.809 and 0.803 for mortality, 0.85 and 0.84 for institutionalization, and 0.86 and 0.85 for homecare initiation, respectively. Internal validity measured by bootstrap method found almost equal estimates.

**Table 5:** C-statistic and pseudo-R^2^ estimates for the outcomes of mortality, institutionalization, and homecare and the concurrent CFS and RISC scales

	Concurrent subsample	1 year		2 years		R^**2**^
		Apparent	Bootstrap	Apparent	Bootstrap	
**Mortality**		0.809	0.809	0.803	0.803	0.11
**Institutionalization**		0.851	0.850	0.841	0.841	0.06
**Homecare**		0.861	0.861	0.853	0.853	0.07
**CFS**	0.821^(*)^					0.21
**RISC**	0.848^(*)^					0.30

*Note*: Adjusted by age and gender. ^(*)^ Area Under the Curve

For the concurrent analysis, AUC were 0.82 and 0.85 for CFS and RISC, respectively. Pseudo-R2 estimates of calibration were low for all outcomes.

**Fig. 2:**
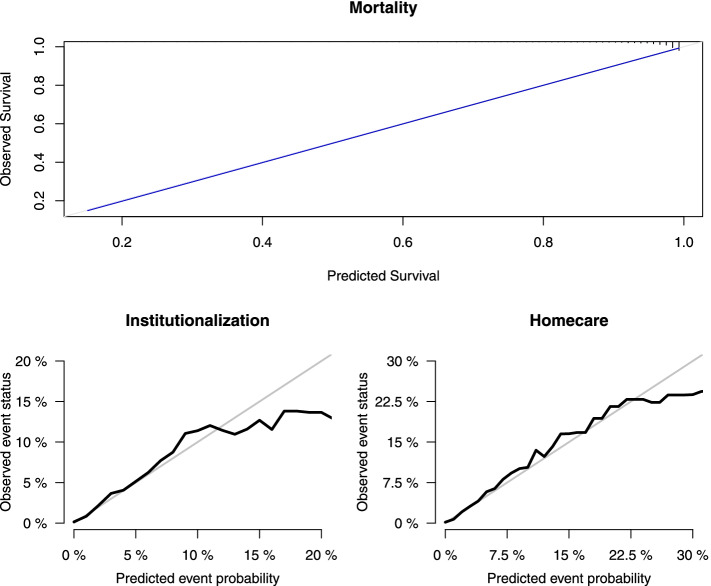
Calibration plots at two years: Predicted survival and Observed frequencies. Mortality, Institutionalization and Homecare outcomes

Figure 2 depicts calibration plots at 2 years for mortality, institutionalization, and homecare outcomes, reflecting agreement between outcome predictions from the model and the observed ones. The observed and predicted probabilities of the models are plotted.

## Discussion

In this study, we have adapted and validated the eFI developed by Clegg et al. [10] using routine electronic data from the Catalan Primary Healthcare records and the SIDIAP database. The new frailty index, eFRAGICAP, was calculated in a EHR cohort of elderly individuals who had a two-year follow-up for adverse outcomes. In addition, we included a baseline random subsample where we independently measured two frailty scales (CFS and RISC) to concurrently validate the data obtained from the SIDIAP database.

It was found that increasing levels of frailty predicted institutionalization and homecare needs, outcomes associated with functional decline and loss of autonomy. Individuals with moderate and severe frailty had over 6 and 10-fold increased risk, respectively, of entering a homecare program or nursing home. Mortality was also predicted, with over 3 and 7-fold increased risk of death for moderate and severe frailty, respectively. The performance of the eFRAGICAP was also as good in the detection of follow-up adverse outcomes as in the identification of frail subjects compared to the CFS (AUC 0.821) and the RISC (AUC 0.848). It demonstrated that both prospective and concurrent validation processes were aligned.

The objective of the eFRAGICAP is to measure and stratify frailty. Many instruments can be found in the literature ranging from frailty phenotypes to the accumulation of deficits [4, 8]. Few, however, are based on electronic data and thus can be automatized [25]. In our cohort from Barcelona, we observed that 59% of the population aged ≥65 years assigned to a PHC had some level of frailty, with 26.5% presenting moderate to severe. A figure similar to the global 57% reported in the external cohort by Clegg et al. although with higher numbers in the moderate/severe categories. In our study, the index average was greater than that described in the British cohort of the original eFI. While this was possibly due to differences between the two populations, for instance, our Mediterranean cohort was slightly older, other possible reasons might include variations in healthcare contexts and database codes. The registration of the electronic data from both the Barcelona cohort and the validation subsample was exhaustive. With respect to the varying deficit prevalence between the two indices, eFI and eFRAGICAP, such differences are not uncommon and mainly attributable to dissimilar populations, healthcare systems, and registry techniques. Nevertheless, only small variations were observed in mean age between the two studies, 75 and 76 in the British and Catalan one, respectively. Our median index was 0.14, higher in women and older subjects, following already described patterns in the literature to date [10, 26]. Other studies measuring frailty in our context are difficult to compare due to differences in the instruments employed. Frailty indices stratify the accumulation of risk conditions registered over time, whereas physical frailty scales and performance tests can measure more precisely signs and symptoms related to functional decline. Using Fried’s criteria, Serra-Prat [27] reported 31% frailty and 49% pre-frailty, though the study population was slightly older. A recent systematic revision [28] on worldwide frailty prevalence with FIs observed a prevalence of 49% for pre-frailty and 24% for frailty. Findings that concur with ours and also agree with data from research in the United States using EHR [29].

The statistical models showed good results at 2 years of follow-up for the categories of mild, moderate, and severe frailty to predict death, institutionalization, and homecare need. The estimates of predictive validity from the global cohort follow-up were very similar to those obtained in the concurrent validation subsample in an independent data collection. In addition, in the two-year prediction of institutionalization and homecare a competing risk analysis was performed to prevent death before outcome from modifying the analysis.

The comparison of eFRAGICAP with the CFS demonstrated that individuals rated as mildly frail or more in the CFS had a mean eFRAGICAP score of 0.36 vs 0.24 of those in the first four CFS categories. Those who scored medium-high risk in the RISC tool had a mean eFRAGICAP score of 0.38 vs 0.23 found in the low-risk subjects. The concurrent multivariate models also showed an growing trend of belonging to the CFS and RISC risk categories as the frailty index increased: adjusted OR for CFS > 4 of 2.41, 9.29, and 16.67, and adjusted OR for RISC of 1.18, 4.09, and 58.72 for mild, moderate, and severe frailty respectively.

The eFRAGICAP has, therefore, been proven to be a good and efficient way to measure frailty. It can be performed easily in primary care without the excessive time consumption implied by face-to face measurements such as performance tests, or frailty phenotypes. Indeed, primary care is the ideal setting to screen for frailty and patients at risk, nevertheless, healthcare professionals require agile tools as consultation time is limited. As has been implemented in the United Kingdom, risk stratification employing routinely collected data can be of considerable use to primary healthcare professionals [11]. Furthermore, frailty indices may also be used in other settings such as nursing homes and hospitals as has been shown in other studies [30, 31].

An additional advantage of eFRAGICAP is that it employs the ICD-10 [18, 19]. The most internationally employed generic system, the ICD-10 can be adapted to the Observational Medical Outcomes Partnership common data model. This will facilitate the subsequent development of an international standard to measure frailty [32].

### Strengths and limitations

A major strength of our study is that it was based on criteria defined by Clegg et al. [10]. They were adapted to the Catalan EHC routinely used by the PHC without requiring additional evaluation as the care program for the elderly homogeneously evaluates their status.

The ICD-10 codes were exhaustively mapped with eFI as codes were provided for the conversion from REDCODE. In addition, the validation was carried out with a stringently studied and analyzed cohort. Another crucial aspect is that the results obtained in the survival models in the area under the curve were > 80%.

Some limitations must be taken into account. There was evidence of good discrimination for the outcomes of mortality, institutionalization, and homecare need. When stratifying for risk, the index performed well, partially due to the fact that the eFRAGICAP incorporates concepts of multimorbidity and associated diseases. Nevertheless, other measures such as physical frailty scales can better differentiate between frailty and disability and are perhaps more specific identifying frailty signs and symptoms. Concurrent validation with the CFS show variations between the two approaches in the classification of frail individuals. Nevertheless, such differences are to be expected as they are based on different theoretical frameworks. The proportion of functional deficits contained within the eFRAGICAP is low compared to other standardized measures of frailty. In contrast, that of diseases and comorbidity has considerable weight.

Despite such classification differences, globally the eFRAGICAP performs well when compared to the CFS and RISC (AUC > 80%).

Such a limitation requires further research to improve the tool, for instance, the inclusion in the EHR of a wider range of functional impairment deficits, and other geriatric scales or performance tests. In addition, the eFRAGICAP, as the eFI, considers each variable to have the same weight even though not all deficits may contribute equally to the outcomes. Further studies are needed to adjust the weight of each deficit to each outcome and take into account other factors such as age and gender.

Moreover, as healthcare systems and clinical health records evolve, codes may be extended and adapted. The index is therefore open to being improved with respect to clinical, demographic, and systemic changes.

## CONCLUSIONS

The main objective of the study was to define an FI with routinely collected data from the EHR which would allow categories with predictive validity for adverse outcomes (mortality, institutionalization, and homecare need) to be established. The index distribution concurs with the literature and presents a good discriminative capacity when compared to other frailty scales. A routine automated, population-based identification and stratification of frailty can be useful in the management of elderly patients in both primary care settings and the community, provided it is associated with effective early interventions.

## Supplementary Information

**Additional file 1.**

## Data Availability

The datasets generated and/or analyzed during the current study are available in the Open Science Framework repository, doi:10.17605/OSF.IO/U6W39, [https://osf.io/u6w39/].

## Abbreviations

AUC: Area Under the Curve
ADLS: Activities of Daily Living
CFS: Clinical Frailty Scale
CIF: Cumulative Incidence Function
eFI: electronic Frailty Index (United Kingdom)
eFRAGICAP: Electronic Frailty Index, primary healthcare centres, Catalunya
EHR: Electronic Health Records
FI: frailty index
ICD-10: International Classification of Diseases, 10th version
ICS: Catalan Health Institute
PHC: primary healthcare centres
RISC: Risk Instrument for Screening in the Community
SHR: Sub-distribution Hazard Risk
SIDIAP: Information System for the Development of Research in Primary Care
