# Supplementary material for: Validation of an electronic frailty index with electronic health records: eFRAGICAP index
Source: BMC Geriatr. 2022 May 7;22:404. doi: 10.1186/s12877-022-03090-8 (PMC9080132; doi:10.1186/s12877-022-03090-8)
Supplement: Supplementary file 1 — Additional file 1. [file 12877_2022_3090_MOESM1_ESM.docx]

# Supplementary tables

| DEFICIT | ICD-10 CODES/Other variables |
| --- | --- |
| Mobility and transfer problems | **Z99.3**  Transfer (0,1)  Stairs (0)  Mobility (0,1)  Timed Get Up&Go: ≥20s or unable |
| Housebound | **Z74.9** Homecare programme  Transfer (0) |
| Activity limitation | **Z73.6, Z63.6**  Barthel index: total <= 90  Law on dependency granted |
| Visual impairment | **H25*, H26*, H28*, H40*, H42*, H53*, H54*** |
| Hearing impairment | **H90*, H91*, Z46.1, Z96.2, Z97.4** |
| Requirement for care | **Z59.3** Nursing home  Home help granted  Has a carer |
| Social vulnerability | **Z63.9 , Z63.2 , Z63.4 , Z60.9 , Z59.1 , Z59.6 , Z73.4 , Z73.5 , Z60.2 , Z60.4 , Z60, Z60.* , Z59 , Z59.0 , Z59.1 , Z59.2 , Z59.4 , Z59.5 , Z59.6 , Z59.7 , Z59.8 , Z59.9 , Z60.2 , Z73.91 , Z91.81 , Z91.2**  Social Risk Indicators (TIRS;>0),  Socio-Family Rating Scale of the Elderly (SFRSE;>=10),  Older Americans Resources and Services (OARS; >18),  Social worker involved |
| Falls | **W0*, W1*, R29.6 , R26.89**  Has fallen in the last year  Number of falls in the last year  Provision of telecare alarm service |
| Urinary incontinence | **R32, N39.3, N39.4**  I13211 (urinary incontinence, stress),  I13212 (urinary incontinence, reflex),  I13213 (urinary incontinence, urge),  I13214 (urinary incontinence, functional),  I13215 (urinary incontinence, total) |
| Weight loss and anorexia | **R63.0, R63.3, R63.4, R64, E46**  Mini Nutritional Assessment (MNA-SF®; ≤11)  BMI < 18.5 |
| Memory and cognitive problems | **F00*, F01*, F02*, F03*, F06.7, G30*, G31*, R41.81**  I831 (memory impairment)  Lobo Mini Cognitive Examination (MCE; ≤ 23)  Short Portable Mental State Questionnaire (SPMSQ; >2) |
| Dizziness | **R42*, H81*, H82*** |
| Dyspnoea | **R06.0**  Dyspnoea increase (Anthon criteria, 1)  Dyspnoea Scale from the Medical Research Council (BMRC; 1-4) |
| Polypharmacy | Five or more active invoice |
| Sleep disturbance | **F51, G47.0**  I621 (insomnia) |
| Anaemia and haematinic deficiency | **D46*, D50*, D51*, D52*, D53*, D55*, D56*, D57*, D58*, D59*, D60*, D61*, D62*, D63*, D64***  Haemoglobin, <12g/dl |
| Hypertension | **I10, I11, I11.0, I11.0, I12, I12.0, I12.9, I13, I13.0, I13.1, I13.2, I13.9, I15, I15.0, I15.1, I15.2, I15.8, I15.9**  Systolic and Diastolic pressure (≥140/90) |
| Ischaemic heart disease | **I20*, I21*, I22*, I23*, I24*, I25*** |
| Heart failure | **I11.0, I13.0, I13.2, I50***  New York Heart Association classification (NYHA, class II- III) |
| Cerebrovascular disease | **G45*, G46*, I60*, I61*, I62*, I63*, I64*, I65*, I66*, I67*, I68*, I69*, S06.6, S06.5** |
| Peripheral vascular disease | **I70*, I71, I71.2, I71.4, I71.9, I72, I72.9, I73, I73.0, I73.9, I74, I74.9, I77, I77.0, I77.6, I77.9, E10.5, E11.5, E13.5**  Ankle brachial pressure index (ABPI ≤0.9) |
| Atrial fibrillation | **I48*** |
| Heart valve disease | **I05*, I06*, I07*, I08*, I33*, I34*, I35*, I36*, I37*, I38*, I39*** |
| Hypotension/syncope | **I95*, R55** |
| Diabetes | **E10*, E11*, E12*, E13*, E14*, H36.0, G59.0, G63.2, H28.0, M14.2, N08.3, E08.6** |
| Foot problems | **L84** |
| Arthritis | **M0*, M1*, M42*, M45*, M46, M47, M48, M77.2, Z96.6** |
| Respiratory disease | **J42*, J43*, J44*, J45*, J46**  Need for domiciliary oxygen |
| Peptic ulcer | **K25*, K26*, K27*, K28*** |
| Thyroid disease | **D34*, E01*, E02*, E03*, E04*, E05*, E06*, E07*, R94.6**  Thyroid stimulating hormone level (TSH abnormal levels, <0.4->5) |
| Chronic kidney disease | **N18*, N19*, Q61*, E10.2, E11.2, E13.2, E14.2**  Glomerular filtration rate (<60) |
| Osteoporosis | **M80*, M81*, M82***  Hip T-Score (<-2.5) |
| Fragility fracture | **M80*, M84*, M48.4, M48.5, M9.5, S12*, S22*, S32*, S52*, S62*, S72*, T08, T08.0, T10.0** |
| Parkinsonism and tremor | **G20*, G21*, G22*, G25*, R25.1** |
| Urinary system disease | **N0*, N1, N2*, N30*, N31*, N32*, N33*, N34*, N35*, N36*, N37*, N38*, N39, N39.9** |
| Skin ulcer | **I83.0, I89.0, L97**  Braden Scale assessment (BRADEN≤12) |

Table S1. ICD-10 codes, clinical and laboratory measures that were considered to create each of the 36 frailty deficits. The inclusion of * after an ICD-10 code (appear in bold) means that all the codes that derive from the main code are included.

| Deficit | eFRAGICAP | eFI [1] |
| --- | --- | --- |
| Hypertension  Polypharmacy  Arthritis  Urinary system disease  Visual impairment  Respiratory disease  Requirement for care  Diabetes  Fragility fracture  Osteoporosis  Urinary incontinence  Social vulnerability  Peripheral vascular disease  Anaemia  Falls  Sleep disturbance  Activity limitation  Hearing impairment  Mobility & transfer problems  Chronic kidney disease  Dyspnoea  Thyroid disease  Memory & cognitive problems  Cerebrovascular disease  Atrial fibrillation  Ischaemic heart disease  Heart valve disease  Housebound  Hypotension/syncope  Heart failure  Parkinsonism & tremor  Dizziness  Peptic ulcer  Skin ulcer  Weight loss & anorexia  Foot problems | 63.00  55.50  44.60  34.30  34.00  28.20  27.20  22.80  19.40  19.30  18.90  18.40  18.30  18.00  17.90  16.60  16.10  14.00  13.70  13.20  13.20  13.10  11.20  10.60  9.80  9.70  6.80  6.60  6.30  6.10  5.50  3.70  3.60  3.30  2.90  0.50 | 50.30  69.00  32.60  29.10  26.80  24.80  6.00  14.20  8.20  8.10  5.40  7.40  4.30  23.20  10.10  6.90  1.10  16.10  5.80  20.80  14.80  14.20  4.70  9.30  8.80  18.30  0.80  13.70  8.00  4.90  1.30  14.90  5.30  4.40  3.50  2.50 |

Table S2. Prevalence of each frailty deficit. eFRAGICAP refers to the prevalence of the codes in the population of study using the coding from Table S1. eFI reports the prevalence of each deficit in the population from the Clegg et al. (2016) [1] study using their coding.

# References

1. Clegg A, Bates C, Young J *et al.* Development and validation of an electronic frailty index using routine primary care electronic health record data. *Age Ageing* 2016;**45**:353–60.
